# Fruit Quality Characteristics and Biochemical Composition of Fully Ripe Blackberries Harvested at Different Times

**DOI:** 10.3390/foods10071581

**Published:** 2021-07-07

**Authors:** Maja Mikulic-Petkovsek, Robert Veberic, Metka Hudina, Zala Zorenc, Darinka Koron, Mateja Senica

**Affiliations:** 1 Chair for Fruit Growing, Viticulture and Vegetable Growing, Department of Agronomy, Biotechnical Faculty, University of Ljubljana, Jamnikarjeva 101, SI-1000 Ljubljana, Slovenia; robert.veberic@bf.uni-lj.si (R.V.); metka.hudina@bf.uni-lj.si (M.H.); 2 Agricultural Institute of Slovenia, Hacquetova Ulica 17, SI-1000 Ljubljana, Slovenia; zala.zorenc@kis.si (Z.Z.); darinka.koron@kis.si (D.K.); 3 Department of Food Science and Technology, Biotechnical Faculty, University of Ljubljana, Jamnikarjeva 101, SI-1000 Ljubljana, Slovenia; mateja.senica@bf.uni-lj.si

**Keywords:** blackberry fruits, quality parameters, fruit weight, fruit colour, sugars, organic acids, phenolic compounds, ripening season

## Abstract

We investigated how the quality of blackberry fruit changes during the ripening period. Since it is difficult to predict picking dates for blackberries, we were interested in how the quality of fully ripe fruit changed depending on the sampling date (from 28 July to 1 September). Blackberries (at full ripeness) were sampled at six weekly intervals and the contents of sugars, vitamin C, organic acids and phenolic components were analysed by high performance liquid chromatography combined with mass spectrometry. The colour parameters, total soluble solids and weight of the fruits were also measured. The results showed that the fruits at the last sampling had a significantly lower fruit weight and higher soluble solids. ‘Cacanska Bestrna’ had the highest fruit weight and vitamin C content (11.43 mg/100 g). The main sugars in blackberries were fructose (13.8–33.4 g/kg FW) and glucose (13.0–33.2 g/kg FW). ‘Loch Ness’ and ‘Navaho’ had a sweeter taste since they had the highest ratio of sugars and acids (S/A approx. 5.8) and ‘Smoothstem’ and ‘Thornfree’ had the sourest taste, with a ratio of S/A 2.5. Blackberries harvested at the first two samplings had lower anthocyanin contents than fruits from later sampling times. There were no significant differences in the content of flavonols, ellagitannins, flavanols or hydroxycinnamic acids during the ripening period. The content of vitamin C in the fruits did not change among samplings, but the fruits had a higher content of organic acids at the first two or three samplings. The results may be useful for both the processing industry and growers to set quality standards for each variety and to determine the optimal harvest time.

## 1. Introduction

Blackberries (*Rubus fruticosus* L.) are grown all over the world, but areas with mild winters and long temperate summers are more suitable for their growth. The main areas of blackberry production are North America, Europe, Asia, South America, Central America and Africa. Many high-quality and high yield varieties are used today in blackberry production [1].

Blackberries are present on the market for the purpose of fresh consumption; however, more often, they are processed into juices, jams, purees, concentrates and sweets. The quality of the fruit is extremely important for the consumer and the food industry, both the external appearance of the fruit and the internal quality, which is directly related to the content of primary and secondary metabolites. It is also important to remember that high-quality fruit has a higher market value [2].

Sugars and organic acids are the main water-soluble substances in berries and have a great influence on the taste and ripeness of blackberry fruits, and even represent an index of acceptability for the consumer. Organic acids, sugars and their ratios, together with various secondary and aromatic compounds, play an important role in the taste and organoleptic properties of fruit [3]. Organic acids also reduce the growth of microorganisms in fruit juices and, consequently, have an impact on better preserving the quality of products. In fruits, they are usually found in free form; they are rarely bound by cations (potassium, sodium). Organic acids also help stabilise anthocyanins, and ascorbic acid plays an important role in extending the shelf life of berries [4]. Immature fruits have higher acid contents and, as they ripen, their content decreases sharply [5].

The popularity and acceptability of a particular type of fruit among consumers is not only because of their high nutritional value, characteristic taste and aroma, but also because of their positive health-promoting properties [6]. Blackberries are a good source of vitamins, minerals and other bioactive compounds, such as phenolics. In recent years, an increasing trend in demand for foods high in high phenolic content has been observed [7], mainly because of their antioxidant properties [6]. Blackberries are extremely rich in bioactive substances: anthocyanins, ellagitannins, flavonol glycosides and phenolic acids, which contribute to their high antioxidant potential [8,9,10]. Clinical studies have shown that eating fruit that contains high concentrations of anthocyanins, as well as some other phenolic groups, can reduce the risk of obesity, cardiovascular disease, degenerative diseases and various forms of cancer [11,12,13].

Growing conditions, ripening process and the degree of ripeness are the key factors influencing fruit taste [14]. The onset and duration of ripening of blackberry fruits depends on the cultivar and environmental factors. They are characterised by successive ripening, which is a problem in relation to fruit picking, since it is not possible to harvest the whole yield at once. Blackberry fruits have the best quality and also the best taste when they are fully mature. Based on colour alone, it is difficult to determine the optimal picking time. In practice, this is when the shiny dark colour changes to a dull colour and the fruits are easily plucked. Blackberry fruits are very delicate, so they should be picked in the cold part of the day and then immediately stored in a refrigerator [15,16].

The purpose of our study was to investigate how the quality parameters of blackberries change depending on different harvest times, since the fruits ripen progressively. Our second goal was to try to determine the optimal harvest time for each blackberry cultivar, at which the fruits have the highest quality. In reviewing the literature, we found that several experiments have been made to study the different degrees of ripeness of blackberry fruits. However, there are scarce data on changes in the chemical composition of optimally ripened fruits at different harvest times. The experiment included five blackberry cultivars (‘Cacanska Bestrna’, ‘Loch Ness’, ‘Navaho’, ‘Smoothstem’ and ‘Thornfree’), which were always harvested at full fruit ripeness at six different times throughout the season. The purpose of the study was to determine the differences in fruit weight, colour parameters, soluble solids, content of vitamin C, sugars, organic acids and phenolic compounds between different harvest times. Producers and the food industry can use the content of nutrients in blackberries as a criterion for determining the internal quality of the crop.

## 2. Materials and Methods

### 2.1. Plant Materials

‘Cacanska Bestrna’, ‘Loch Ness’, ‘Smoothstem’ and ‘Thornfree’ blackberry varieties were planted in 2007 and ‘Navaho’ in 2013 in the experimental station of the Agricultural Institute of Slovenia in central Slovenia. Plants were trained on a trellis with supporting wires. The space between plants in the row was 1.3 m. Each plant had three to five canes. During the summer, canes were topped and lateral branches were cut back. With pruning, we keep the plant canopy from becoming too vigorous and ensured optimal fruit ripening. Because of successive ripening in a full season, we harvested once per week to pick ripe fruit. All fruits were harvested on the outside part of the plant canopy. Ripe fruits were black, glossy and easily picked. Ripe fruits of all blackberry varieties were picked during the week ending on: 28 July, 4 August, 10 August, 18 August, 25 August and 1 September.

### 2.2. Measurements of Fruit Colour

The colour of the blackberry fruit was measured with a portable colorimeter (Konica Minolta CR-10 Plus Color Reader, Tokyo, Japan). The apparatus was adjusted with a reference white porcelain tile before the determination. Measured colour parameters were lightness L* (ranges from 0—black to 100—reference white), chroma C* (colour saturation or intensity) and hue angle h° (colour hue). The values for h° were from 0 to 360°, where 0° is red, 90° is yellow, 180° is green and 270° is blue/purple [17]. Colour parameters were measured on 30 blackberry fruits at each sampling for each cultivar.

### 2.3. Determination of Fruit Weight, pH of Juice and Total Soluble Solids (TSS)

At each sampling, five fruits of each blackberry cultivar were weighed together in 10 replicates. From the obtained weight, we calculated the average weight for one fruit. The pH value and the content of soluble solids (TSS) were measured in the juice obtained by squeezing several blackberry fruits. Soluble solids were determined with a digital hand-held refractometer (30 PX, Mettler Toledo, Greifensee, Switzerland) and values expressed in °Brix. The pH value of the juice was measured with a pH meter (inoLab pH/Cond 720, WTW, Xylem analytics, Weilheim, Germany). Ten replications of measurements were performed for each cultivar for each individual sampling.

### 2.4. Extraction and Analysis of Sugars and Organic Acids

For the extraction of sugars and acids, 2 g of blackberry fruits were weighed and soaked in 8 ml of bidistilled water and mashed with a homogeniser (Ultra-Turrax, Ika Labortechnik, Staufen, Germany). The samples were extracted for 30 minutes at 20 °C and constantly shaken [3]. The extracts were then centrifuged and filtered through 0.20 μm cellulose mixed esterspore filters (Chromafil PP/MV A-20/25, Macherey-Nagel, Düren, Germany) into glass vials. Samples were analysed by high performance liquid chromatography (HPLC; Finnigan Surveyor, Thermo Fischer Scientific, San Jose, CA, USA). Determination of sugars was performed with a Rezex RCM-monosaccharide Ca + (2%) (Phenomenex, Torrance, CA, USA) column heated to 65 °C using an RI detector, and the mobile phase was bidistilled water with a flow rate of 0.6 ml/min. Organic acids were analysed on the same HPLC system equipped with a UV detector (210 nm), the column was Rezex ROA-organic acids H + (8%) (Phenomenex, Torrance, CA, USA), heated to 65 °C; the mobile phase was 4 mM sulfuric acid. Sugars and organic acids were determined by comparing retention times with standards. The contents of sugars and organic acids were expressed in mg/100 g of fresh blackberries.

### 2.5. Extraction and Analysis of Vitamin C

The fruits were crushed into a smooth pulp in a mortar cooled with liquid nitrogen, and 3 grams of fruits were extracted with 9 mL of 2% meta-phosphoric acid [18]. The extraction was carried out for half an hour at a temperature of 4 degrees Celsius. The extracts were then centrifuged and filtered through 0.20 μm cellulose mixed esterspore filters (Chromafil PP/MV A-20/25, Macherey-Nagel, Düren, Germany) into vials. For the analysis of vitamin C, we used a UV detector set at 210 nm and a Rezex ROA column − organic acids H + (8%) (Phenomenex), which was heated to 20 °C. The mobile phase used was 4 mM sulfuric acid and a flow rate of 0.6 mL/min. The vitamin C content was expressed in mg/100 g of fresh blackberries.

### 2.6. Extraction and Analysis of Phenolic Components

A quantity of 2.5 g of crushed blackberries was weighed and poured over with 8 mL of methanol containing 3% formic acid. The samples were extracted for 50 min in an ice-cold ultrasonic bath. The extracts were then centrifuged and filtered through polyamide nylonpore filters (Chromafil PP/PA AO-20/25, Macherey-Nagel, Düren, Germany) into vials. Phenolic compounds were analysed on an HPLC system (Thermo Fischer Scientific, Accela, San Jose, CA, USA) equipped with a DAD detector. It was measured at 280 nm, 350 nm (flavonols, flavanols, hydroxybenzoic and hydroxycinnamic acids) and at 530 nm for anthocyanins. A Gemini C18 column operating at 25 °C was used for analysis. Individual phenolic compounds were analysed by mixing two mobile phases: A was 0.1% formic acid and 3% acetonitrile in bidistilled water and B was 0.1% formic acid and 3% water in acetonitrile. All phenolic compounds were identified by comparing UV-VIS spectra and retention times with standards and confirmed using a mass spectrometer (Thermo Fisher Scientific, San Jose, USA, Thermo Finningan LCQ Deca XP MAX LC/MSn), with an electrospray interface (ESI) operating in the negative and positive ion range [18]. On the chromatogram, we integrated the peak areas of the identified phenolic compounds and recalculated the peak areas in blackberry samples with the corresponding standards. The phenolic content of blackberries was expressed in mg/100 g fresh weight.

### 2.7. Statistical Analysis

All measurements and contents are given as mean ± standard error of 10 replicates. Significant differences between sampling dates and between blackberry cultivars were calculated using two-way analysis of variance (ANOVA) with the statistical program R-Commander (R Formation for Statistical Computing, Auckland, New Zealand). Differences in all measurements and nutrient contents were evaluated using Duncan’s multiple range test. *p* values of less than 0.05 were considered statistically significant.

## 3. Results and Discussion

### 3.1. Fruit Colour, Weight and pH of Juice and Total Soluble Solids

We used five blackberry cultivars (‘Cacanska Bestrna‘, ‘Loch Ness’, ‘Navaho’, ‘Smoothstem’ and ‘Thornfree’) in the experiment, which are interesting because of their high yield and the good chemical quality of the fruits. All the studied cultivars are suitable for fresh use or freezing, as well as processing. Throughout the ripening period of blackberries, we sampled the fruits at six weekly intervals and monitored the fruit quality parameters. Fruit weight, dry soluble matter, juice pH and colour parameters (L, C and H) were determined. For all samplings, we also performed a chemical analysis of the fruit, by which we determined the content of individual sugars, organic acids, vitamin C and the content of phenolic substances.

In general, the fruit weight was lowest toward the end of the season (Table 1). Fruit weight decreased by 25% (‘Thornfree’) to 44% (‘Cacanska Bestrna’) in relation to the period in which fruits had the highest fruit weight. The reason for the decrease in berry weight is due to the greater transpiration from the fruit and the impeded phloem flow when the berry reaches its maximum weight [19]. The cultivar with the highest average fruit weight was ‘Cacanska Bestrna’ (8.4 g). Previous studies have also reported that ‘Cacanska Bestrna’ is distinguished by its high fruit weight [20].

Among the visual parameters, the colour of the fruit is crucial for a buyer in deciding to buy fruit. Consumers want blackberries with extremely dark purple fruits and a shiny appearance, since a dull colour can give the fruit the appearance of either freshness or excessive fruit age [21,22]. The colour of blackberries depends on various factors, such as genotype, production conditions, fruit ripening stage, harvesting time, climate and soil, as well as storage conditions [23,24]. The measured colour parameter h ° in blackberries ranged between 346 and 351, which means a dark purple colour, while the L* value was from 19.8 to 23.9, which gives the fruit an extremely dark, almost black colour (Table 1). However, a shiny fruit appearance is a result of the chrome parameter (C*), which ranged from 22.7 to 25.5. The higher the chrome values are, the more intense the colour of the fruit and, consequently, the more attractive to the consumer [25]. There were no significant differences in the measured parameters L*, C* and h ° among the different blackberry cultivars, and only minor differences among different sampling times. The values of the colour parameters were comparable to the literature [26].

In the measured pH values of blackberry juice, we found some differences between individual samplings (Table 1), but it is not possible to come to any conclusion regarding the period of harvest during the season at which the pH is higher or lower. There were no significant differences in the pH of the juices among the cultivars. The pH values of blackberry juice are quite low compared to other fruit species, ranging from 2.7 to 3.1. However, significant differences in the content of total soluble solids were shown, since the fruits of all cultivars had a significantly higher TSS at the last sampling (1 September) (Table 1). The exceptions were ‘Thornfree’, with which we measured the highest TSS at sampling 3 (10 August) and sampling 6 (1 September) and cultivar ‘Smootstem’, which had the highest TSS at samplings 5 (25 August) and 6 (1 September). This result is certainly because the fruit weight was lower at the last sampling period and the juice was more concentrated, so its TSS was higher. Interestingly, ‘Loch Ness’ (12.2 °Brix) and ‘Navaho’ (11.5 °Brix) stood out in terms of maximum TSS. A similar finding was reported by Milosevic et al. [19]. However, the fruits at our location achieved slightly higher TSS values than those reported for the same varieties by Milosevic et al. [27].

### 3.2. The Content of Sugars and Organic Acids

The results of soluble solids content are in accordance with the sugar content in blackberry fruits, so the picture is the same; ‘Loch Ness’ (57.0 g/kg FW) and ‘Navaho’ (52.9 g/kg FW) had a significantly higher content of total sugars than other cultivars in the experiment (Table 2). There were no big differences in the sugar content between the individual samplings, so we cannot report a common trend of whether blackberries from the later dates might have significantly more or less sugar. The main sugars in fruits were glucose and fructose, which were approximately in a ratio of 1:1 and represented about 90 to 96% of total sugars. The rest was sucrose (0.1 to 3.4 g/kg). Since fructose is typically sweeter than glucose and sucrose, it is desirable that fruits have a high fructose content, since most consumers prefer sweet fruit [3]. Fructose contents in blackberry fruits ranged from 13.8 to 33.4 g/kg and glucose from 13.0 to 33.2 g/kg FW. Similar sugar values were reported by Veberic et al. [24]. Milivojevic et al. [8] reported slightly higher glucose and fructose contents in blackberries, which is certainly a reflection of different environmental conditions, site locations and technological measures in blackberry production.

Blackberry fruits have a characteristic sour taste, which is due to the low ratio of sugars and acids. The cultivars ‘Smoothstem’ and ‘Thornfree’ had a fairly low sugar/acid ratio, about 2.5, so their fruits had a rather sour taste compared to the cultivars ‘Cacanska Bestrna’, ‘Loch Ness’ and ‘Navaho’, which had a higher S/A ratio, above 3.8, and, therefore, a sweeter taste (Table 2). The following acids were analysed in the blackberry fruits: citric, malic, tartaric, fumaric and shikimic (Table 3). The blackberries contained the highest level of citric acid, which represented as much as 40–50% of all organic acids. Fumaric acid was present in the blackberries only in traces. The content of total acids in the cultivars ‘Loch Ness’, ‘Navaho’ and ‘Smoothstem’ was significantly higher in the first two and three sampling periods, while the level of acids did not differ between the cultivars ‘Cacanska Bestrna’ and ‘Thornfree’. ‘Thornfree’ and ‘Smoothstem’ cultivars stand out for the highest acid content (12.8 and 13.7 g/kg FW). The analysed acid values in blackberries are comparable to previous studies [16], while [28] reported higher concentrations of acids in wild blackberries from southern Bulgaria. We assume that wild blackberry accessions contain higher acid contents. 

Analysis of vitamin C showed that the vitamin C content of the blackberry fruits did not change significantly among harvest times (Table 3). The exception was the first sampling with ‘Navaho’, when the fruits had significantly the lowest vitamin C content. In terms of high vitamin C content, ‘Cacanska Bestrna’ stood out, with 11.4 mg of vitamin C 100 g, which was a 50% higher content than ‘Thornfree’, which had the lowest vitamin C content (7.5 mg/100 g). The content of vitamin C in fruits is certainly genetically determined, so there are big differences among individual genotypes. An approximately two times higher vitamin C content has been reported in wild blackberries, i.e., from 20.4 to 28.1 mg/100g [23]. Similar values of vitamin C to our results have been reported in the cultivars ‘Jumbo’, ‘Blacksatin’ and ‘Dirksen’ (7.1 to 9.6 mg/100 g) [29]. In addition to genotype, the location of the site and all its environmental conditions (climate, weather, soil etc.), as well as the season and maturity stage, contribute to the content of vitamin C in fruits [7].

### 3.3. The Content of Phenolic Compounds

The contents of secondary metabolites, especially phenolic compounds, also play an important role in fruit quality. Fruit colour is the main factor of fruit quality and makes the product attractive to the customer, so colour also determines the market value of fruit [2]. Anthocyanins are the most important phenolic group in blackberries due to their high content, since they represented approximately 50–70% of all phenolics analysed. Of the anthocyanins, we analysed six cyanidin derivatives (cyanidin-3-glucoside, cyanidin-3-rutinoside, cyandin-3-arabinoside, cyanidin-3-xyloside, cyanidin-3-(6″malonylglucoside) and cyanidin-3-(6″-dioxalylglucoside)) and one pelargonidin derivative (pelargonidin-3-glucoside). The content of total cyanidin glycosides ranged from 527 to 914 mg/kg of fruit (Table S1), while the content of pelargonidin-3-glucoside was very low, only 5.4 to 12.6 mg/kg. The analysed anthocyanin contents are comparable to previous publications [10]. Despite the fact that the presence of cyanidin derivatives in fruit is reported to contribute to a red coloration [30], the reason for the dark blackberry coloration is an extremely high concentration of cyanidin glycosides. It is the same with elderberries and dark carrots, whose fruits are reflected by a very dark purple, almost black colour, but contain mainly cyanidin derivatives [31,32].

**Figure 1:**
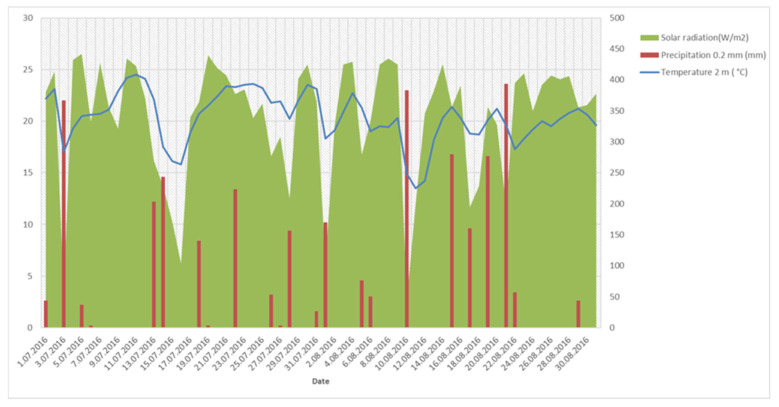
Variation of temperature, precipitation and solar radiation from 1 July to 31 August.

However, the colour of the fruit is also known to be affected by the pH of the juice. A major storage problem with blackberries is that when the fruit is frozen, the colour changes from black to red. This is probably because anthocyanins pass from the vacuole contents into the cytoplasma of cells. Above all, this problem is noticeable when immature blackberry fruits are picked, which are apparently ripe and dark, but still have insufficient anthocyanin content. Anthocyanins are then diluted in the cytoplasma, which causes a change in the colour of the fruit or discoloration from black to red of the frozen fruits [15]. In our study, when we sampled fully ripe blackberry fruits several times during the ripening season, the results showed that the fruits had a significantly lower content of anthocyanins at the first sampling (‘Navaho’) or at the first two samplings (‘Cacanska Bestrna’, ‘Thornfree’ and ‘Smoothstem’) compared to later sampling times. Environmental factors, especially sunlight, UV light and higher day temperatures, make a strong contribution to anthocyanin synthesis [33,34]. At the later sampling times, i.e., from 5 August onwards, the blackberry fruits ripened faster, due to the higher sum of solar radiation hours, as well as the higher solar intensity (Figure 1). Although better lighting results in increased anthocyanin synthesis in fruits, extremely high exposure to sunlight and high temperatures can result in decreased anthocyanin concentrations in fruits [35]. The ‘Loch Ness’ cultivar had, on average, the highest anthocyanin content (863 mg/kg), while ‘Cacanska Bestrna’ had the lowest anthocyanin content (614 mg/kg).

In addition to anthocyanins, other phenolic groups were also analysed in fruits: flavanols represented 10–25% of the total analysed phenolic content, flavonol glycosides 5–10%, ellagitannins 5–15% and phenolic acids up to 2%. From the group of flavanols, catechin, epicatechin and different procyanidins were analysed (Table S2). The total flavanol content ranged from 99.9 to 335.2 mg/kg of fresh blackberries. By monitoring the flavanol content through weekly samplings, we found that it is not possible to reach a common conclusion about the trend of changes in their content. It can perhaps be said that the fruits at the first sampling (‘Cacanska Bestrna’) or at the first three samplings (‘Loch Ness’ and ‘Navaho’) had higher flavanol contents than the fruits from later samplings. This result may indicate that the fruits were not yet fully ripe, since it is known that immature or less ripe fruits contain higher levels of catechin and epicatechin compared to ripe fruits [36]. ‘Cacanska Bestrna’ had a 1.5 to 2.2 times lower content of total flavanols (120.4 mg/kg FW) compared to the other studied cultivars, which would mean that its fruits have a less astringent taste.

Elagitannins (ETs) are compounds in which gallic acid is bound to hexahydroxydiphenic acid (HHDP). We distinguish among monomeric (derivatives of ellagic acid), oligomeric (Sanguiin H-6) and polymeric forms of ellagitannins. During their hydrolysis, ellagic acid is released. ETs together with gallotannins belong to the group of hydrolysing tannins [37]. Elagitannins have been found in various berries, e.g., raspberries, strawberries, blueberries and cloudberries [38]. In our experiment, total ellagitannin contents in blackberries ranged from 43.4 to 202.3 mg/kg FW (Table S3). ‘Smoothstem’ and ‘Thornfree’, with an average content of 181.5 and 189.4 mg/kg FW, respectively, stood out for their high content of ellagitannins. Interestingly, ‘Loch Ness’ had up to 3.5 times less total ellagitannins at all sampling times. There were only slight differences in their content between the different weekly sampling dates. In general, the content of ellagitannins in ripe blackberries was quite stable. Since ellagitannins have many positive effects on human health, it is recommended to include raspberries, strawberries and blackberries in the daily diet. Research results show that ellagitannins have antioxidant, anti-microbial and anti-inflammatory effects, as well as probiotic effects [11,39].

Flavonols are a highly desirable group of secondary metabolites and interest in them has been increasing recently, mainly due to their beneficial pharmaceutical properties, such as anticarcinogenic, antioxidant and anti-inflammatory [12,13]. Various flavonol glycosides were identified in the blackberries: eight quercetin glycosides, three kaempferol (galactoside, glucoside and rutinoside) and two isorhamnetin derivatives (glucuronide and rhamnoside) (Table S3). Quercetin glycosides are the main phenolic group, representing approximately 60% of the total flavonols in cultivars ‘Cacanska Bestrna’ and ‘Smoothstem’, 80% in cultivars ‘Navaho’ and ‘Loch Ness’ and even 95% in ‘Thornfree’ fruits. From 63.7 to 81.7 mg of quercetin glycosides per kilogram were found in the blackberries. Kaempferol derivatives appeared in much lower contents, i.e., from 2.6 to 7.8 mg/kg, and isorhamnetin derivatives from 4.3 to 15.6 mg/kg, which represented about 5–15% of total flavonols. Isorhamnetin derivatives were not confirmed in ‘Thornfree’ (Table S3). No significant fluctuations in total flavonol levels in the mature fruits were observed between sample periods from 28 July to 1 September. Other publications have also reported that flavonol content steadies in the state of ripe fruits [9]. ‘Cacanska Bestrna’ (115.4 mg/kg FW) and ‘Smoothstem’ (122 mg/kg FW) had a significantly higher content of total flavonols during the study period than the other cultivars. A similar range of flavonol content was reported in six American blackberry genotypes, ranging from 99–150 mg/kg FW [40].

Among the phenolic compounds formed in berries, hydroxycinnamic acids (HCA) also play an important role due to their diversity and content. In the synthetic pathway, they are formed from cinnamic acid, and their basic structure is usually from caffeic, *p*-coumaric, ferulic, syringic and sinapic acid [41]. A fairly low content of hydroxycinnamic acids, 4.5 to 22.2 mg/kg (Table S3), was found in ripe blackberry fruits. In ‘Navaho’, however, we did not identify any HCA representatives. Previous research also reported the absence of phenolic acids in the fruits of some blackberry varieties [10]. A significant change in HCA content between dates was not found.

**Figure 2:**
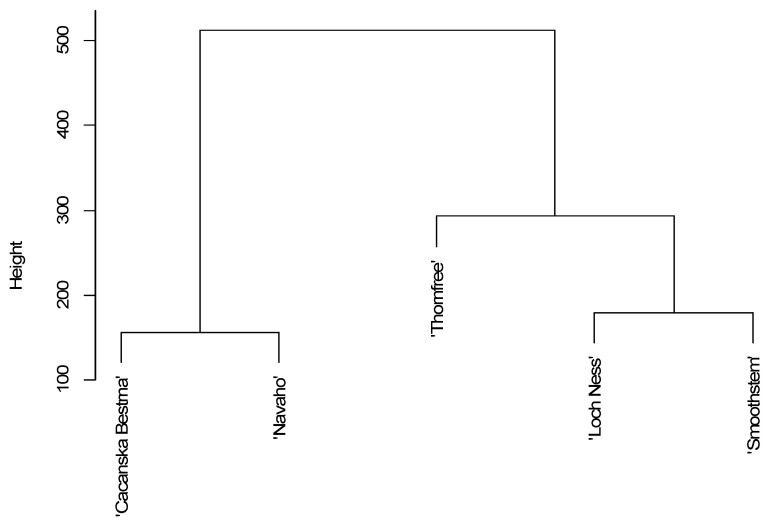
Dendrogram of the analysed primary and secondary metabolites among different blackberry cultivars, using Ward’s method, based on the square Euclidean distance.

Dendrogram clustering showed some dissimilarity among the different blackberry cultivars (Figure 2). It was characterised by three distinct branches, with ‘Cacanska Bestrna’ and ‘Navaho’ in one cluster, ‘Loch Ness’ and ‘Smoothstem’ in a second and ‘Thornfree’ in a third. The ‘Thornfree’ blackberry cultivar had the highest contents of all analysed compounds, while the cultivars ‘Cacanska Bestrna’ and ‘Navaho’ from the first cluster had the lowest contents of both primary and secondary metabolites. ‘Loch Ness’ and ‘Smoothstem’ from the second cluster had similar contents of all analysed primary and secondary metabolites. Their contents were higher than those of ‘Cacanska Bestrna’ and ‘Navaho’ cultivars from the first cluster. The blackberry cultivar ‘Thornfree’ was the highest, mainly due to some analysed secondary metabolites and organic acids of primary metabolites.

## 4. Conclusions

Blackberries are an extremely important source of beneficial substances for health. They are available to the consumer throughout the whole year, since most of the crop is frozen or processed. It is certainly important for production, however, that a yield is achieved that has the best external and internal (phytochemical content) fruit quality. For blackberries, it is difficult to predict the optimal time to harvest them. We were, therefore, interested in how the quality of fully ripe blackberries varies depending on the picking date (from 28 July to 1 September). We found that there were no very drastic changes in the content of chemical substances (flavanols, flavonols, elagitannins, HCA) among the sampling periods. It was noticeable, however, that the blackberries had a higher content of organic acids and a lower content of anthocyanins at the first and sometimes at the second sampling, which can probably be attributed to the fact that the fruits were not yet fully ripe despite their ripe appearance. At the last sampling, lower fruit weight and the highest soluble solid content were determined. ‘Thornfree’ had the highest level of total phenolic contents (almost 1600 mg/kg) (Table S4). The highest contributors to them were total anthocyanins and ellagitannins. The richest in elagitannins were ‘Thornfree’ and ‘Smoothstem’, while the richest in anthocyanins was ‘Loch Ness’ (863 mg/kg). From the point of view of growers, as well as the processing industry, the results of a detailed chemical analysis of blackberries can be used as a basis for determining fruit quality criteria based on their chemical content.

## Figures and Tables

**Table 1:** The content of different main fruit parameters (mean ± standard error) of blackberry fruits and a two-way ANOVA of the harvest date, cultivar and the interaction between the harvest date × cultivar.

Cultivar	Term	TSS			pH			L			C			h			Fruit Weight		
			± se	s.		± se	s.		± se	s.		± se	s.		± se	s.		± se	s.
‘Cacanska Bestrna’	T1	9.26	0.24	b	2.77	0.03	cd	23.62	0.25	a	24.26	0.76	ab	346.90	0.22	a	10.08	0.07	a
	T2	6.90	0.11	d	2.71	0.03	d	22.74	0.68	a	23.92	0.60	ab	348.96	0.95	a	8.62	0.09	ab
	T3	8.26	0.34	c	2.95	0.02	ab	23.36	0.28	a	23.42	0.76	b	348.08	0.85	a	9.07	0.41	b
	T4	7.36	0.15	d	3.08	0.09	a	19.86	1.75	b	25.24	0.32	ab	348.22	0.63	a	9.44	0.20	b
	T5	9.72	0.17	b	2.93	0.05	ab	23.60	0.34	a	25.42	0.52	a	348.28	0.53	a	7.14	0.48	c
	T6	10.52	0.21	a	2.87	0.06	bc	22.36	0.80	ab	24.48	0.53	ab	348.48	0.72	a	5.95	0.18	d
‘Loch Ness’	T1	11.10	0.25	c	2.79	0.03	bc	23.86	0.25	a	24.36	0.89	ab	347.48	1.11	b	5.55	0.41	bc
	T2	10.58	0.22	c	2.75	0.07	c	23.46	0.35	a	24.40	0.49	ab	347.34	0.28	b	5.81	0.13	b
	T3	13.32	0.28	b	3.12	0.06	a	22.52	0.41	a	23.32	0.31	b	351.40	0.68	a	6.90	0.09	a
	T4	11.00	0.07	c	3.07	0.04	a	20.62	0.66	b	25.34	0.41	a	348.10	1.03	b	6.74	0.18	a
	T5	12.90	0.13	b	2.91	0.02	b	23.08	0.57	a	25.48	0.36	a	348.40	0.62	b	4.85	0.12	c
	T6	14.34	0.22	a	3.11	0.04	a	23.30	0.17	a	24.10	0.15	ab	348.28	0.80	b	5.35	0.24	bc
‘Navaho’	T1	11.24	0.27	bc	2.81	0.02	b	23.28	0.63	a	23.30	1.30	a	348.30	1.09	a	5.70	0.11	b
	T2	11.42	0.22	b	3.04	0.05	a	23.76	0.56	a	24.58	0.61	a	348.66	1.01	a	5.54	0.09	bc
	T3	11.46	0.26	b	3.03	0.04	a	21.94	1.11	ab	24.68	0.41	a	348.76	0.71	a	7.29	0.37	a
	T4	10.74	0.11	c	3.08	0.04	a	19.78	1.47	b	24.84	0.64	a	349.28	1.12	a	7.45	0.19	a
	T5	11.82	0.15	b	2.96	0.02	a	22.44	0.35	ab	24.04	0.17	a	349.36	0.63	a	6.33	0.25	b
	T6	12.30	0.26	a	3.00	0.02	a	23.96	0.55	a	24.92	0.26	a	347.20	0.62	a	4.84	0.34	c
‘Smoothstem’	T1	7.96	0.15	b	2.74	0.02	d	23.48	0.26	a	22.66	1.65	a	349.56	1.20	a	6.56	0.48	a
	T2	7.92	0.12	b	2.76	0.02	d	23.34	0.43	a	24.90	0.37	a	348.00	0.52	a	6.17	0.47	ab
	T3	8.60	0.13	b	2.81	0.02	cd	23.90	0.38	a	24.76	0.14	a	348.70	1.01	a	6.05	0.13	ab
	T4	7.98	0.20	b	2.98	0.04	a	20.50	1.29	b	24.56	0.61	a	348.44	1.61	a	6.63	0.22	a
	T5	9.66	0.12	a	2.88	0.28	bc	23.92	0.50	a	24.96	0.21	a	347.86	0.46	a	5.41	0.29	bc
	T6	9.90	0.47	a	2.93	0.03	ab	23.04	0.52	a	24.10	0.58	a	349.26	1.04	a	4.82	0.07	c
‘Thornfree’	T1	8.40	0.18	b	2.82	0.02	ab	23.12	0.62	ab	23.86	1.16	ab	347.68	1.03	b	6.75	0.18	ab
	T2	7.68	0.18	c	2.83	0.03	ab	23.32	0.27	ab	24.54	1.16	ab	346.08	1.01	b	6.13	0.15	bc
	T3	9.42	0.30	a	2.78	0.18	b	22.42	0.94	ab	21.56	1.08	b	351.76	0.90	a	6.33	0.26	bc
	T4	7.84	0.15	bc	3.07	0.04	a	21.46	0.47	b	25.32	0.81	a	348.66	0.84	ab	7.07	0.18	a
	T5	8.24	0.19	bc	3.03	0.02	ab	24.08	0.70	a	24.94	0.33	a	348.98	1.47	ab	5.71	0.23	cd
	T6	8.96	0.11	a	3.02	0.03	ab	21.84	0.34	b	23.68	1.10	ab	349.66	1.35	ab	5.26	0.17	d
Cultivar	‘Cacanska Bestrna’	8.67	0.25	C	2.91	0.07	A	22.90	0.95	A	24.64	0.93	A	347.88	1.49	A	8.38	0.35	A
	‘Loch Ness’	12.21	0.27	A	2.95	0.08	A	22.42	1.24	A	24.03	1.88	A	348.65	2.87	A	5.87	0.19	B
	‘Navaho’	11.50	0.12	B	2.85	0.11	A	21.80	1.14	A	24.15	0.86	A	349.20	1.28	A	6.19	0.24	B
	‘Smoothstem’	8.67	0.18	C	2.83	0.09	A	23.18	1.28	A	24.38	0.78	A	348.25	1.72	A	5.94	0.19	B
	‘Thornfree’	8.42	0.13	C	2.94	0.10	A	22.26	2.09	A	24.33	2.36	A	348.58	3.25	A	6.21	0.16	B
	*p* term	0.0000 ***			1.2985			33.25			2.905			11.438			0.000 ***		
	*p* cultivar	0.0000 ***			0.5842			2.29			9.941			4.219			0.000 ***		
	*p* INT	0.0017 **			0.0173			2.42			2.562			4.557			0.000 ***		

^1^ Different small letters (a–d) in the columns denote statistically significant differences between the sampling dates for each blackberry cultivar by Duncan’s multiple range test (*p* < 0.05). Different capital letters (A–C) in columns denote statistically significant differences by Duncan’s multiple range test (*p* < 0.05) among different cultivars (** statistically significant differences at a *p*-value < 0.001; *** statistically significant differences at a *p*-value < 0.0001; no asterisk—statistically non-significant). TSS is the total soluble solids (°Brix), fruit weight is in grams. Harvest dates: T1—28.07; T2—4.08; T3—10.08; T4—18.08.; T5—25.08; T6—1.9, all in 2016.

**Table 2:** The content of individual and total sugars (mean ± standard error in g/kg FW) of blackberry fruits and two-way ANOVA of harvest date, cultivar and interaction between harvest date × cultivar.

Cultivar	Term	Fructose			Glucose			Sucrose			Total Sugars		
			± se			± se			± se			± se	
‘Cacanska Bestrna’	T1	20.25	1.49	a	19.46	1.48	a	2.53	0.84	a	42.23	2.58	a
	T2	13.76	0.58	b	13.02	0.48	b	1.80	0.79	a	28.58	1.80	b
	T3	18.20	0.90	a	17.60	0.89	a	3.15	0.51	a	38.95	1.54	a
	T4	18.60	1.27	a	17.64	1.17	a	2.55	0.28	a	38.79	2.21	a
	T5	20.23	1.20	a	19.57	1.28	a	2.26	0.26	a	42.06	2.29	a
	T6	20.61	0.95	a	19.87	0.94	a	2.01	0.35	a	42.49	2.13	a
‘Loch Ness’	T1	24.87	1.52	c	24.34	1.51	c	0.50	0.35	ab	49.72	2.71	d
	T2	25.09	0.93	c	24.43	0.88	c	0.90	0.42	ab	50.42	1.71	cd
	T3	33.44	1.05	a	33.21	1.19	a	0.11	0.03	b	66.77	2.22	a
	T4	28.51	0.77	b	27.73	0.85	b	0.13	0.05	b	56.38	1.58	b
	T5	27.50	0.50	bc	26.94	0.52	bc	1.14	0.32	a	55.58	0.74	bc
	T6	31.71	0.48	a	31.32	0.62	a	0.25	0.04	b	63.29	1.05	a
‘Navaho’	T1	24.10	0.77	b	23.46	0.84	b	1.38	0.18	a	48.94	1.54	b
	T2	26.86	1.02	ab	26.23	1.02	ab	0.99	0.17	a	54.08	1.99	ab
	T3	28.48	1.75	a	27.92	1.77	a	1.16	0.40	a	57.56	3.23	a
	T4	26.47	1.11	ab	25.43	1.36	ab	1.04	0.07	a	52.95	2.44	ab
	T5	25.57	1.56	ab	24.42	1.64	ab	1.01	0.03	a	51.00	3.20	ab
	T6	26.50	0.97	ab	25.76	0.94	ab	0.79	0.09	a	53.05	1.94	ab
‘Smoothstem’	T1	13.81	1.19	c	13.02	1.22	c	3.39	0.51	a	30.21	2.67	b
	T2	15.33	0.21	bc	14.69	0.21	bc	1.76	0.27	b	31.79	0.38	b
	T3	17.11	0.75	ab	16.36	0.74	ab	1.85	0.21	b	35.32	1.31	ab
	T4	15.63	0.72	bc	14.86	0.76	bc	1.10	0.33	b	31.60	1.41	b
	T5	19.92	0.40	a	19.06	0.37	a	2.11	0.36	b	41.09	1.10	a
	T6	18.99	1.60	a	18.31	1.55	a	1.79	0.40	b	39.09	3.25	a
‘Thornfree’	T1	14.40	0.82	b	13.73	0.81	b	2.87	0.19	a	31.00	1.45	b
	T2	13.47	0.30	b	12.87	0.26	b	2.35	0.37	ab	28.69	0.65	b
	T3	22.04	0.87	a	21.37	0.88	a	1.59	0.73	ab	45.00	1.12	a
	T4	13.99	4.38	b	13.10	1.32	b	1.02	0.57	b	28.12	1.90	b
	T5	14.51	0.64	b	13.98	0.48	b	2.30	0.38	ab	30.78	0.98	b
	T6	16.30	0.35	b	15.67	0.50	b	1.86	0.26	ab	33.83	1.04	ab
Cultivar	‘Cacanska Bestrna’	18.61	0.63	C	17.86	0.63	C	2.38	0.22	A	38.85	6.02	C
	‘Loch Ness’	28.52	0.75	A	28.00	0.78	A	0.51	0.02	C	57.02	7.12	A
	‘Navaho’	26.33	0.53	B	25.54	0.56	B	1.06	0.08	B	52.93	5.15	B
	‘Smoothstem’	16.80	0.55	CD	16.05	0.55	CD	2.00	0.19	A	34.85	5.38	C
	‘Thornfree’	15.78	0.91	D	15.12	0.91	D	2.00	0.21	A	32.90	8.86	C
	*p* term	0.0000 ***			0.000 ***			0.000 ***			0.000 ***		
	*p* cultivar	0.0000 ***			0.000 ***			0.000 ***			0.000 ***		
	*p* INT	0.0017 **			0.000 ***			0.000 ***			0.000 ***		

^1^ Different small letters (a–d) in columns denote statistically significant differences between sampling dates for each blackberry cultivar by Duncan’s multiple range test (*p* < 0.05). Different capital letters (A–C) in columns denote statistically significant differences by Duncan’s multiple range test (*p* < 0.05) among different cultivars (** statistically significant differences at a *p*-value < 0.001; *** statistically significant differences at a *p*-value < 0.0001; no asterisk—statistically non-significant). Harvest dates: T1—28.07; T2—4.08; T3—10.08; T4—18.08.; T5—25.08; T6—1.9, all in 2015.

**Table 3:** The content of individual and total organic acids (mean ± standard error in g/kg; fumaric and shikimic acid in mg/100 g) and vitamin C (mean ± standard error in mg/100 g FW) of blackberry fruits and two-way ANOVA of harvest date, cultivar and interaction between harvest date × cultivar.

Cultivar	Term	Citric Acid			Malic Acid			Tartaric Acid			Fumaric Acid			Shikimic Acid			Total Organic Acids			Vitamin C		
			± se	*s.*		± se	*s.*		± se	*s.*		± se	*s.*		± se	*s.*		± se	*s.*		± se	*s.*
‘Cacanska Bestrna’	T1	4.79	0.30	a	4.36	0.46	a	2.66	0.20	a	0.13	0.10	b	2.27	0.07	a	11.84	0.91	a	10.27	0.80	a
	T2	5.28	0.31	a	4.07	0.41	a	2.70	0.15	a	0.24	0.02	ab	2.10	0.14	a	12.07	0.83	a	11.16	0.84	a
	T3	5.28	0.22	a	5.14	0.39	a	2.19	0.03	a	0.36	0.03	a	2.24	0.09	a	12.63	0.48	a	11.22	0.77	a
	T4	4.70	0.41	a	4.80	0.61	a	1.91	0.23	a	0.45	0.03	a	2.41	0.05	a	11.44	1.18	a	11.81	0.82	a
	T5	4.45	1.35	a	3.99	1.25	a	2.13	0.64	a	0.39	0.11	a	1.86	0.56	a	10.58	3.22	a	11.85	0.67	a
	T6	3.48	1.13	a	2.86	0.85	a	1.88	0.66	a	0.40	0.08	a	1.72	0.44	a	8.24	2.64	a	12.25	0.45	a
‘Loch Ness’	T1	6.10	0.32	a	2.16	0.65	a	3.29	0.22	a	0.82	0.09	a	1.05	0.10	ab	11.58	0.28	a	8.75	0.75	a
	T2	6.03	0.22	a	3.02	0.20	a	3.29	0.20	a	0.84	0.09	a	1.19	0.13	a	12.36	0.59	a	9.85	0.46	a
	T3	4.00	0.23	bc	2.18	0.16	a	2.04	0.24	b	0.76	0.11	a	1.03	0.03	ab	8.25	0.63	b	9.19	0.67	a
	T4	3.61	0.93	c	2.26	0.13	a	2.44	0.15	b	0.48	0.04	b	1.03	0.29	ab	8.32	0.83	b	9.39	0.46	a
	T5	5.14	0.09	ab	2.94	0.21	a	3.13	0.11	a	0.60	0.04	ab	1.00	0.04	ab	11.23	0.36	a	9.22	0.95	a
	T6	4.66	0.25	bc	2.06	0.08	a	2.53	0.16	b	0.67	0.04	ab	0.61	0.26	b	9.27	0.46	b	7.84	0.88	a
‘Navaho’	T1	4.85	0.17	a	3.79	0.02	a	2.73	0.11	a	0.35	0.02	a	1.70	0.04	abc	11.38	0.25	a	4.52	0.77	c
	T2	4.31	0.15	ab	3.44	0.09	ab	2.46	0.07	ab	0.41	0.01	a	1.92	0.08	a	10.23	0.28	ab	8.49	0.45	b
	T3	4.12	0.28	ab	3.71	0.29	a	2.15	0.13	c	0.38	0.02	a	1.75	0.05	ab	10.00	0.68	ab	8.93	0.41	ab
	T4	2.94	0.78	b	3.56	0.07	ab	2.17	0.07	bc	0.27	0.09	a	1.54	0.08	bc	8.69	0.70	bc	8.89	0.15	ab
	T5	2.93	0.80	b	3.60	0.12	a	2.35	0.09	bc	0.34	0.02	a	1.62	0.09	bc	8.91	0.81	bc	8.79	0.48	b
	T6	4.17	0.25	ab	3.12	0.14	b	2.65	0.09	a	0.29	0.09	a	1.50	0.09	c	7.57	0.46	c	10.32	0.30	a
‘Smoothstem’	T1	7.15	0.30	a	5.68	0.38	a	3.31	0.23	a	0.41	0.01	a	2.78	0.10	b	16.16	0.89	a	10.17	0.40	a
	T2	6.33	0.25	b	4.77	0.35	a	2.96	0.11	a	0.46	0.17	a	2.50	0.25	b	14.08	0.66	ab	10.69	0.27	a
	T3	6.38	0.10	b	4.99	0.19	a	2.98	0.11	a	0.43	0.21	a	2.77	0.14	b	14.38	0.38	ab	10.64	1.59	a
	T4	5.23	0.22	c	4.77	0.43	a	2.40	0.16	a	0.22	0.14	a	2.66	0.06	b	12.43	0.80	b	9.98	0.68	a
	T5	6.10	0.23	b	3.73	1.26	a	2.18	0.73	a	0.16	0.09	a	2.35	0.27	a	12.04	1.79	b	9.79	1.11	a
	T6	5.23	0.24	c	4.61	0.26	a	3.01	0.27	a	0.18	0.13	a	2.73	0.12	b	12.89	0.73	b	7.96	0.22	a
‘Thornfree’	T1	6.07	0.12	a	4.15	1.02	a	2.54	0.07	a	0.55	0.04	a	2.39	0.08	b	12.79	0.98	a	6.73	0.30	ab
	T2	6.25	0.30	a	5.37	0.36	a	2.62	0.13	a	0.35	0.13	ab	2.26	0.07	b	14.26	0.67	a	8.04	0.61	ab
	T3	5.63	0.25	a	4.61	0.49	a	2.41	0.25	a	0.24	0.14	ab	2.47	0.14	ab	12.67	0.86	a	8.55	0.96	ab
	T4	5.47	0.20	a	5.01	0.61	a	2.46	0.21	a	0.36	0.13	ab	2.43	0.08	ab	12.97	1.02	a	5.53	0.87	b
	T5	4.24	0.41	a	4.73	0.57	a	2.28	0.75	a	0.38	0.11	ab	2.77	0.17	a	11.27	1.72	a	6.69	0.66	ab
	T6	4.83	0.31	a	5.26	0.35	a	2.47	0.14	a	0.14	0.00	b	2.18	0.11	b	12.58	0.73	a	9.21	1.45	a
Cultivar	‘Cacanska Bestrna’	4.66	0.30	C	4.20	0.30	A	2.24	0.16	B	0.33	0.03	B	2.10	0.12	C	11.13	0.73	B	11.43	0.30	A
Cultivar	‘Loch Ness’	4.92	0.25	BC	2.44	0.14	B	2.79	0.12	A	0.69	0.04	A	0.99	0.07	E	10.17	0.39	BC	9.04	0.29	BC
Cultivar	‘Navaho’	3.89	0.23	D	3.14	0.25	B	2.42	0.06	AB	0.34	0.02	B	1.67	0.04	D	9.47	0.33	C	8.34	0.41	CD
	‘Smoothstem’	6.07	0.16	A	4.76	0.25	A	2.80	0.15	A	0.31	0.06	B	2.80	0.08	A	13.66	0.46	A	9.87	0.37	B
	‘Thornfree’	5.42	0.27	AB	4.85	0.32	A	2.46	0.13	AB	0.34	0.05	B	2.42	0.06	B	12.76	0.64	A	7.46	0.44	D
	*p* term	0.0000 ***			0.000 ***			0.005 **			0.1743			0.0448 *			0.005 **			0.0148 *		
	*p* cultivar	0.0000 ***			0.0622.			0.004 **			0.000 ***			0.000 ***			0.000 ***			0.000 ***		
	*p* INT	0.294			0.1477			0.550			0.0423 *			0.2131			0.7239			0.0018 **		

Different small letters (a–d) in columns denote statistically significant differences between sampling dates for each blackberry cultivar by Duncan’s multiple range test (*p* < 0.05). Different capital letters (A–C) in columns denote statistically significant differences by Duncan’s multiple range test (*p* < 0.05) among different cultivars (* statistically significant differences at a *p*-value < 0.05; ** statistically significant differences at a *p*-value < 0.001; *** statistically significant differences at a *p*-value < 0.0001; no asterisk—statistically non-significant). Harvest dates: T1—28.07; T2—4.08; T3—10.08; T4—18.08.; T5—25.08; T6—1.9, all in 2015.

## Data Availability

Data are available at corresponding author on reasonable request.

## Supplementary Materials

The following are available online at https://www.mdpi.com/article/10.3390/foods10071581/s1, Table S1: The content of cyanidin glycosides, pelargonidin glycosides and total anthocyanins of blackberry fruits and two-way ANOVA of harvest date, cultivar and interaction between harvest date × cultivar. Table S2: The content of catechin, epicatechin, procyanidin dimers, procyanidin trimers and total flavanols of blackberry fruits and two-way ANOVA of harvest date, cultivar and interaction between harvest date × cultivar. Table S3: The content of ellagitannins, isorhamnetin glycosides, kaempferol glycosides, quercetin glycosides, total flavonols and hydroxycinnamic acids of blackberry fruits and two-way ANOVA of harvest date, cultivar and interaction between harvest date × cultivar. Table S4: The content of total analysed phenolics of blackberry fruits and two-way ANOVA of harvest date, cultivar and interaction between harvest × cultivar.
