# Supplementary material for: Fruit Quality Characteristics and Biochemical Composition of Fully Ripe Blackberries Harvested at Different Times
Source: Foods. 2021 Jul 7;10(7):1581. doi: 10.3390/foods10071581 (PMC8304799; doi:10.3390/foods10071581)
Supplement: Supplementary file 1 [file foods-10-01581-s001.zip › foods-1274623-supplementary.pdf]

Table S1: The content of cyanidin glycosides, pelargonidin glycosides and total anthocyanins (mean ± standard error in mg/kg FW) of blackberry fruits and two-way ANOVA of harvest date, cultivar and interaction between harvest date x cultivar.

| Cultivar | Term | *ANTHOCYANINS* | | | | | | | | |
|---|---|---|---|---|---|---|---|---|---|---|
| | | **Cyanidin glycosides** | | | **Pelargonidin glycosides** | | | **TOTAL** | | |
| | | | ± *se* | *s.* | | ± *se* | *s.* | | ± *se* | *s.* |
| 'Cacanska Bestrna' | T1 | **527.97** | 53.53 | bc | **8.95** | 0.34 | ab | **510.16** | 39.79 | b |
| | T2 | **490.65** | 13.25 | c | **8.48** | 0.44 | c | **499.13** | 13.65 | b |
| | T3 | **640.00** | 29.59 | ab | **10.88** | 0.49 | a | **650.88** | 30.05 | a |
| | T4 | **684.67** | 32.14 | a | **11.11** | 0.24 | a | **695.78** | 32.36 | a |
| | T5 | **676.13** | 31.80 | a | **11.06** | 0.38 | a | **687.19** | 32.05 | a |
| | T6 | **632.71** | 52.89 | ab | **10.66** | 0.57 | a | **643.38** | 53.46 | a |
| | | | | | | | | | | |
| 'Loch Ness' | T1 | **833.49** | 36.75 | a | **7.72** | 1.51 | b | **841.21** | 37.92 | a |
| | T2 | **821.98** | 63.73 | a | **8.78** | 0.91 | ab | **830.76** | 64.63 | a |
| | T3 | **914.30** | 38.90 | a | **9.24** | 0.58 | ab | **923.54** | 38.94 | a |
| | T4 | **887.42** | 41.27 | a | **11.12** | 0.70 | a | **898.54** | 41.94 | a |
| | T5 | **816.42** | 38.56 | a | **10.45** | 0.82 | ab | **826.86** | 39.25 | a |
| | T6 | **851.12** | 8.94 | a | **10.14** | 0.70 | ab | **861.26** | 9.44 | a |
| | | | | | | | | | | |
| 'Navaho' | T1 | **671.15** | 18.85 | b | **5.47** | 1.82 | b | **676.62** | 20.51 | b |
| | T2 | **758.76** | 13.14 | a | **11.77** | 0.60 | a | **770.54** | 13.45 | a |
| | T3 | **790.73** | 25.80 | a | **9.13** | 1.05 | ab | **799.87** | 26.14 | a |
| | T4 | **743.51** | 38.97 | ab | **5.49** | 2.52 | b | **749.00** | 39.28 | ab |
| | T5 | **767.22** | 28.12 | a | **12.56** | 0.35 | a | **779.78** | 28.47 | a |
| | T6 | **743.74** | 25.76 | ab | **10.43** | 0.75 | a | **754.17** | 26.49 | ab |
| | | | | | | | | | | |
| 'Smoothstem' | T1 | **548.85** | 19.56 | d | **8.45** | 0.34 | c | **557.29** | 19.63 | c |
| | T2 | **628.51** | 27.64 | cd | **12.67** | 2.31 | b | **641.18** | 29.94 | bc |
| | T3 | **702.43** | 29.78 | bc | **11.09** | 0.93 | bc | **712.51** | 50.24 | b |
| | T4 | **744.20** | 29.06 | b | **11.41** | 0.94 | bc | **755.61** | 29.40 | b |
| | T5 | **881.71** | 25.22 | a | **18.58** | 1.12 | a | **900.29** | 25.62 | a |
| | T6 | **746.36** | 54.52 | b | **9.62** | 0.76 | bc | **753.48** | 56.84 | b |
| | | | | | | | | | | |
| 'Thornfree' | T1 | **569.95** | 19.15 | b | **5.37** | 1.68 | c | **575.32** | 20.40 | b |
| | T2 | **603.53** | 18.74 | b | **9.79** | 1.62 | bc | **613.33** | 17.51 | b |
| | T3 | **742.11** | 14.81 | a | **11.31** | 0.22 | b | **753.42** | 40.97 | a |
| | T4 | **732.88** | 35.37 | a | **7.44** | 0.24 | bc | **740.31** | 36.91 | a |
| | T5 | **766.61** | 13.13 | a | **16.03** | 1.31 | a | **782.64** | 14.27 | a |
| | T6 | **730.48** | 15.02 | a | **10.29** | 0.72 | b | **740.77** | 41.59 | a |
| | | | | | | | | | | |
| Cultivar | 'Cacanska Bestrna' | **608.69** | 20.61 | C | **10.19** | 0.31 | AB | **614.42** | 22.67 | C |
| | 'Loch Ness' | **854.12** | 16.63 | A | **9.57** | 0.41 | AB | **863.69** | 16.88 | A |
| | 'Navaho' | **745.85** | 12.26 | B | **9.14** | 0.77 | B | **755.00** | 12.65 | B |
| | 'Smoothstem' | **708.68** | 25.38 | B | **11.55** | 0.94 | A | **720.23** | 26.00 | B |
| | 'Thornfree' | **690.93** | 19.11 | B | **10.04** | 0.88 | AB | **700.97** | 19.62 | B |

| | | | | | | | | | | |
|---|---|---|---|---|---|---|---|---|---|---|
| | *p* term<br>*p* cultivar<br>*p* INT | **0.000****<br>**0.000*****<br>**0.0069**** | | | **0.0174****<br>**0.000*****<br>**0.000***** | | | **0.000***<br>**0.000*****<br>**0.0104*** | | |

Different small letters (a-d) in columns denote statistically significant differences between sampling dates for each blackberry cultivar by Duncan's multiple range test ($p<0.05$). Different capital letters (A-C) in columns denote statistically significant differences by Duncan’s multiple range test ($p < 0.05$) among different cultivars. (Asterisk: *- statistically significant differences at p-Value <0.05; ** - statistically significant differences at p-Value <0.001; *** - statistically significant differences at p-Value <0.0001, no asterisk – statistically non-significant). Harvest date: T1- 28.07; T2-4.08; T3-10.08; T4-18.08.; T5-25.08; T6-1.9.2015.

Table S2: The content of catechin, epicatechin, procyanidin dimers, procyanidin trimers and total flavanols (mean ± standard error in mg/kg FW) of blackberry fruits and two-way ANOVA of harvest date, cultivar and interaction between harvest date x cultivar.

| Cultivar | Term | ***FLAVANOLS*** | | | | | | | | | | | | | | |
|---|---|---|---|---|---|---|---|---|---|---|---|---|---|---|---|---|
| | | **Catechin** | | | **Epicatechin** | | | **Procyanidin dimers** | | | **Procyanidin trimers** | | | **TOTAL** | | |
| | | | ± *se* | *s.* | | ± *se* | *s.* | | ± *se* | *s.* | | ± *se* | *s.* | | ± *se* | *s.* |
| 'Cacanska Bestrna' | T1 | | | | **103.15** | 8.82 | a | **16.28** | 2.22 | ab | **14.16** | 1.43 | b | **166.09** | 10.02 | a |
| | T2 | | | | **72.70** | 4.51 | b | **14.36** | 1.99 | b | **13.20** | 3.90 | b | **99.98** | 7.40 | c |
| | T3 | | | | **70.61** | 4.70 | b | **18.85** | 0.46 | ab | **29.20** | 4.00 | ab | **118.05** | 3.28 | bc |
| | T4 | | | | **66.90** | 3.45 | b | **15.03** | 0.82 | b | **21.99** | 7.70 | b | **103.92** | 9.95 | c |
| | T5 | | | | **60.38** | 7.16 | b | **19.59** | 2.86 | ab | **24.72** | 7.74 | b | **104.69** | 11.70 | c |
| | T6 | | | | **67.03** | 3.66 | b | **24.72** | 5.16 | a | **43.79** | 4.90 | a | **135.54** | 8.44 | b |
| | | | | | | | | | | | | | | | | |
| 'Loch Ness' | T1 | **8.86** | 0.83 | ab | **208.13** | 17.88 | ab | **59.87** | 5.28 | a | **28.41** | 6.51 | a | **305.27** | 22.09 | ab |
| | T2 | **9.64** | 1.33 | ab | **225.28** | 26.06 | a | **71.66** | 9.33 | a | **28.57** | 6.71 | a | **335.15** | 30.85 | a |
| | T3 | **11.06** | 0.83 | a | **146.83** | 17.01 | bc | **60.21** | 6.32 | a | **19.81** | 7.37 | a | **237.91** | 30.71 | ab |
| | T4 | **10.02** | 0.86 | a | **131.00** | 11.50 | c | **59.94** | 4.41 | a | **10.55** | 1.78 | a | **211.51** | 18.18 | b |
| | T5 | **7.07** | 0.72 | b | **174.52** | 29.59 | abc | **58.26** | 9.08 | a | **18.39** | 5.80 | a | **258.24** | 35.95 | ab |
| | T6 | **9.41** | 0.29 | ab | **144.82** | 6.84 | bc | **58.22** | 3.92 | a | **17.64** | 2.95 | a | **230.10** | 10.26 | b |
| | | | | | | | | | | | | | | | | |
| 'Navaho' | T1 | | | | **247.77** | 15.70 | a | **31.52** | 2.44 | ab | **16.24** | 1.03 | a | **295.52** | 17.88 | a |
| | T2 | | | | **231.02** | 11.08 | ab | **29.69** | 2.65 | abc | **16.04** | 1.24 | a | **276.75** | 14.31 | ab |
| | T3 | | | | **199.60** | 5.61 | ab | **34.01** | 2.11 | a | **16.33** | 0.62 | a | **249.95** | 4.39 | ab |
| | T4 | | | | **189.45** | 13.09 | b | **26.57** | 0.44 | bcd | **14.63** | 1.11 | a | **230.65** | 12.91 | b |
| | T5 | | | | **188.92** | 28.49 | b | **21.92** | 2.73 | d | **13.56** | 1.73 | a | **224.40** | 32.77 | b |
| | T6 | | | | **194.52** | 12.00 | b | **23.71** | 2.04 | cd | **13.04** | 0.94 | a | **231.27** | 14.52 | b |
| | | | | | | | | | | | | | | | | |
| 'Smoothstem' | T1 | | | | **104.60** | 4.45 | ab | **28.04** | 5.17 | a | **28.25** | 5.89 | a | **160.89** | 4.18 | a |
| | T2 | | | | **121.30** | 12.38 | ab | **43.28** | 9.60 | a | **32.84** | 9.55 | a | **197.42** | 33.28 | a |
| | T3 | | | | **131.68** | 8.25 | a | **47.27** | 10.14 | a | **29.92** | 9.62 | a | **208.87** | 21.80 | a |
| | T4 | | | | **91.54** | 9.65 | b | **27.77** | 7.61 | a | **18.53** | 1.77 | a | **137.84** | 15.87 | a |
| | T5 | | | | **114.68** | 12.03 | ab | **35.48** | 8.57 | a | **37.80** | 8.38 | a | **187.96** | 16.92 | a |
| | T6 | | | | **131.86** | 18.71 | a | **40.66** | 5.86 | a | **22.37** | 0.65 | a | **194.90** | 24.98 | a |
| | | | | | | | | | | | | | | | | |
| 'Thornfree' | T1 | **6.00** | 1.86 | b | **82.18** | 7.15 | c | **98.51** | 6.54 | c | **15.69** | 2.82 | c | **202.39** | 15.18 | c |
| | T2 | **8.49** | 0.22 | ab | **121.20** | 14.87 | ab | **132.80** | 9.37 | b | **31.26** | 3.92 | ab | **293.75** | 14.59 | ab |
| | T3 | **11.33** | 0.69 | a | **125.07** | 7.26 | ab | **138.45** | 3.84 | ab | **34.67** | 5.12 | a | **309.52** | 10.66 | a |
| | T4 | **9.34** | 0.57 | a | **97.72** | 8.11 | bc | **130.98** | 3.38 | b | **22.50** | 1.89 | bc | **260.54** | 13.16 | b |
| | T5 | **9.79** | 0.35 | a | **128.94** | 4.36 | a | **153.80** | 3.25 | a | **32.76** | 4.75 | ab | **325.29** | 8.30 | a |
| | T6 | **9.74** | 0.68 | a | **115.10** | 5.87 | ab | **143.21** | 6.99 | ab | **24.45** | 1.79 | abc | **292.19** | 11.95 | ab |
| | | | | | | | | | | | | | | | | |
| Cultivar | 'Cacanska Bestrna' | | | | **73.41** | 3.55 | D | **18.04** | 1.22 | E | **24.51** | 2.91 | AB | **120.44** | 5.35 | C |
| | 'Loch Ness' | **9.34** | 0.40 | A | **171.76** | 10.15 | B | **61.36** | 2.78 | B | **10.56** | 2.62 | BC | **263.03** | 14.33 | A |
| | 'Navaho' | | | | **208.55** | 7.45 | A | **27.90** | 1.19 | D | **14.98** | 0.50 | C | **251.42** | 8.57 | A |
| | 'Smoothstem' | | | | **115.94** | 5.21 | C | **37.08** | 3.28 | C | **28.29** | 3.31 | A | **181.31** | 9.26 | B |

| | | | | | | | | | | | | | | | | |
|---|---|---|---|---|---|---|---|---|---|---|---|---|---|---|---|---|
| | 'Thornfree' | **9.12** | 0.47 | A | **111.70** | 4.64 | C | **132.96** | 4.18 | A | **26.84** | 1.91 | AB | **280.61** | 9.94 | A |
| | *p* term<br>*p* cultivar<br>*p* INT | **0.660**<br>**0.005****<br>**0.075.** | | | **0.000*****<br>**0.0002*****<br>**0.0001***** | | | **0.000*****<br>**0.004****<br>**0.0007***** | | | **0.0004*****<br>**0.1372**<br>**0.0276*** | | | **0.000****<br>**0.005****<br>**0.000***** | | |

Different small letters (a-d) in columns denote statistically significant differences between sampling dates for each blackberry cultivar by Duncan's multiple range test ($p<0.05$). Different capital letters (A-D) in columns denote statistically significant differences by Duncan’s multiple range test ($p < 0.05$) among different cultivars. (Asterisk: *- statistically significant differences at p-Value <0.05; ** - statistically significant differences at p-Value <0.001; *** - statistically significant differences at p-Value <0.0001, no asterisk – statistically non-significant). Harvest date: T1- 28.07; T2-4.08; T3-10.08; T4-18.08.; T5-25.08; T6-1.9.2015.

Table S3: The content of ellagitannins, isorhamnetin glycosides, kaempferol glycosides, quercetin glycosides, total flavonols and hydroxycinnamic acids (mean ± standard error in mg/kg FW) of blackberry fruits and two-way ANOVA of harvest date, cultivar and interaction between harvest date x cultivar.

| Cultivar | Term | **ELLAGITANNINS** | | | ***FLAVONOLS*** | | | | | | | | | | | | ***HCA*** | | |
|---|---|---|---|---|---|---|---|---|---|---|---|---|---|---|---|---|---|---|---|
| | | **TOTAL** | | | **Isorhamnetin glycosides** | | | **Kaempferol glycosides** | | | **Quercetin glycosides** | | | **TOTAL** | | | **TOTAL** | | |
| | | | **± se** | **s.** | | ± *se* | *s.* | | ± *se* | *s.* | | ± *se* | *s.* | | ± *se* | *s.* | | ± *se* | *s.* |
| 'Cacanska Bestrna' | T1 | **173.33** | 5.17 | a | **5.93** | 0.46 | a | **7.61** | 0.57 | ab | **75.91** | 1.08 | a | **129.78** | 5.59 | a | **15.90** | 1.02 | a |
| | T2 | **144.47** | 4.41 | b | **6.31** | 0.55 | a | **7.76** | 0.28 | a | **70.46** | 1.59 | ab | **126.50** | 4.57 | a | **14.04** | 1.21 | a |
| | T3 | **129.87** | 7.94 | bc | **5.50** | 0.56 | ab | **6.78** | 0.64 | ab | **65.88** | 3.50 | ab | **116.09** | 8.01 | ab | **16.41** | 1.29 | a |
| | T4 | **120.14** | 17.20 | bc | **4.31** | 0.52 | b | **5.88** | 0.80 | b | **60.91** | 6.23 | b | **101.71** | 9.52 | ab | **16.35** | 2.05 | a |
| | T5 | **112.44** | 5.77 | c | **5.18** | 0.30 | ab | **6.72** | 0.20 | ab | **64.28** | 3.40 | b | **107.40** | 1.75 | b | **16.16** | 1.48 | a |
| | T6 | **139.23** | 10.07 | bc | **5.29** | 0.29 | ab | **7.74** | 0.55 | a | **67.54** | 2.67 | ab | **115.72** | 3.76 | ab | **15.00** | 1.58 | a |
| | | | | | | | | | | | | | | | | | | | |
| 'Loch Ness' | T1 | **58.30** | 7.58 | a | **12.82** | 1.15 | ab | **4.73** | 0.58 | a | **69.34** | 4.80 | a | **86.88** | 6.34 | a | **12.92** | 2.06 | a |
| | T2 | **57.41** | 3.38 | a | **13.36** | 1.47 | ab | **5.02** | 0.61 | a | **71.50** | 5.93 | a | **89.88** | 7.84 | a | **15.19** | 2.35 | a |
| | T3 | **50.08** | 6.06 | a | **12.96** | 1.62 | ab | **4.45** | 0.40 | a | **76.37** | 9.88 | a | **93.78** | 11.85 | a | **15.08** | 4.81 | a |
| | T4 | **43.35** | 4.01 | a | **10.90** | 1.05 | b | **4.03** | 0.32 | a | **63.21** | 6.86 | a | **78.15** | 8.13 | a | **12.89** | 0.96 | a |
| | T5 | **53.91** | 6.87 | a | **15.63** | 1.05 | a | **5.33** | 0.23 | a | **79.19** | 8.86 | a | **100.15** | 10.12 | a | **11.59** | 1.76 | a |
| | T6 | **58.07** | 4.13 | a | **13.25** | 0.72 | ab | **4.99** | 0.32 | a | **80.99** | 4.29 | a | **99.23** | 5.31 | a | **12.68** | 1.99 | a |
| | | | | | | | | | | | | | | | | | | | |
| 'Navaho' | T1 | **75.68** | 4.55 | a | **13.55** | 0.79 | a | **6.07** | 0.30 | a | **67.25** | 4.12 | a | **86.88** | 4.83 | a | | | |
| | T2 | **77.61** | 4.25 | a | **12.77** | 0.61 | a | **5.50** | 0.16 | ab | **74.58** | 3.34 | a | **92.85** | 3.99 | a | | | |
| | T3 | **69.74** | 3.48 | a | **11.18** | 0.49 | a | **4.52** | 0.20 | c | **71.58** | 3.25 | a | **87.29** | 3.53 | a | | | |
| | T4 | **71.96** | 8.17 | a | **12.14** | 1.64 | a | **4.52** | 0.46 | c | **71.36** | 4.62 | a | **88.03** | 6.46 | a | | | |
| | T5 | **75.37** | 4.85 | a | **12.25** | 0.77 | a | **4.71** | 0.26 | bc | **75.03** | 7.40 | a | **92.00** | 8.32 | a | | | |
| | T6 | **77.07** | 4.34 | a | **12.58** | 0.45 | a | **4.89** | 0.35 | bc | **81.73** | 7.09 | a | **99.20** | 7.67 | a | | | |
| | | | | | | | | | | | | | | | | | | | |
| 'Smoothstem' | T1 | **168.77** | 8.58 | ab | **6.50** | 0.34 | a | **7.17** | 0.45 | a | **68.81** | 1.31 | b | **131.01** | 5.80 | a | **13.91** | 2.23 | a |
| | T2 | **199.28** | 12.64 | a | **5.66** | 0.45 | ab | **6.32** | 0.39 | a | **71.11** | 1.97 | ab | **120.95** | 1.68 | ab | **15.08** | 1.48 | a |
| | T3 | **193.47** | 10.22 | ab | **5.07** | 0.42 | ab | **6.41** | 0.43 | a | **73.59** | 4.24 | ab | **124.28** | 4.08 | ab | **22.20** | 5.69 | a |
| | T4 | **166.50** | 12.06 | ab | **5.33** | 0.37 | ab | **6.79** | 0.58 | a | **67.42** | 1.53 | b | **113.27** | 2.84 | b | **13.83** | 2.89 | a |
| | T5 | **162.12** | 8.67 | b | **5.49** | 0.27 | b | **6.54** | 0.16 | a | **71.33** | 1.71 | ab | **116.26** | 2.41 | b | **18.10** | 2.91 | a |
| | T6 | **198.82** | 9.72 | a | **5.06** | 0.34 | b | **6.92** | 0.38 | a | **80.43** | 4.81 | a | **126.17** | 6.44 | ab | **13.99** | 2.77 | a |
| | | | | | | | | | | | | | | | | | | | |
| 'Thornfree' | T1 | **200.79** | 12.33 | a | | | | **4.28** | 0.97 | a | **63.68** | 6.11 | b | **67.96** | 7.06 | b | **4.46** | 0.88 | b |
| | T2 | **202.26** | 10.03 | a | | | | **3.70** | 0.13 | ab | **74.66** | 2.70 | ab | **78.36** | 2.71 | ab | **8.22** | 0.35 | a |
| | T3 | **188.05** | 11.40 | a | | | | **3.13** | 0.37 | ab | **78.81** | 2.99 | a | **81.94** | 3.17 | a | **9.92** | 0.97 | a |
| | T4 | **166.71** | 4.36 | a | | | | **2.57** | 0.18 | b | **65.42** | 2.94 | b | **67.99** | 3.06 | b | **8.89** | 1.01 | a |
| | T5 | **187.60** | 6.77 | a | | | | **3.02** | 0.27 | ab | **70.68** | 1.86 | ab | **73.70** | 1.80 | ab | **8.73** | 0.85 | a |
| | T6 | **191.10** | 9.67 | a | | | | **3.22** | 0.25 | ab | **74.61** | 4.93 | ab | **77.83** | 5.15 | ab | **9.35** | 0.55 | a |
| | | | | | | | | | | | | | | | | | | | |
| Cultivar | 'Cacanska Bestrna' | **136.58** | 5.34 | B | **5.42** | 1.04 | B | **7.08** | 0.24 | A | **67.50** | 1.61 | A | **115.36** | 3.03 | A | **15.64** | 0.65 | AB |
| | 'Loch Ness' | **53.52** | 2.30 | D | **13.15** | 2.57 | A | **4.76** | 0.18 | B | **73.43** | 2.85 | A | **91.34** | 3.47 | B | **13.39** | 0.57 | B |
| | 'Navaho' | **74.57** | 1.96 | C | **12.41** | 1.73 | A | **5.04** | 0.16 | B | **73.59** | 2.11 | A | **91.04** | 2.37 | B | | | |
| | 'Smoothstem' | **181.49** | 5.02 | A | **5.52** | 0.82 | B | **6.69** | 0.16 | A | **72.12** | 1.38 | A | **121.99** | 1.98 | A | **16.18** | 1.34 | A |

| | | | | | | | | | | | | | | | | | | | |
|---|---|---|---|---|---|---|---|---|---|---|---|---|---|---|---|---|---|---|---|
| | 'Thornfree' | **189.42** | 4.83 | A | | | | **3.32** | 0.20 | C | **71.31** | 1.79 | A | **74.63** | 1.88 | C | **8.26** | 0.47 | C |
| | *p* term | **0.000*** ** | | | **0.000*** ** | | | **0.000*** ** | | | **0.1760** | | | **0.000*** ** | | | **0.000*** ** | | |
| | *p* cultivar | **0.0001*** ** | | | **0.0542.** | | | **0.000*** ** | | | **0.0083** ** | | | **0.0131*** | | | **0.109** | | |
| | *p* INT | **0.0458*** | | | **0.3706** | | | **0.6284** | | | **0.6692** | | | **0.5462** | | | **0.613** | | |

Different small letters (a-d) in columns denote statistically significant differences between sampling dates for each blackberry cultivar by Duncan's multiple range test ($p<0.05$). Different capital letters (A-D) in columns denote statistically significant differences by Duncan's multiple range test ($p < 0.05$) among different cultivars. (Asterisk: *- statistically significant differences at *p*-Value <0.05; ** - statistically significant differences at *p*-Value <0.001; *** - statistically significant differences at *p*-Value <0.0001, no asterisk – statistically non-significant). Harvest date: T1- 28.07; T2-4.08; T3-10.08; T4-18.08.; T5-25.08; T6-1.9.2015.

Table S4: The content of total analyzed phenolics (mean ± standard error in mg/kg FW) of blackberry fruits and two-way ANOVA of harvest date, cultivar and interaction between harvest x cultivar.

| Cultivar | Term | Total analyzed phenolics | | |
|---|---|---|---|---|
| | | | | |
| | | | ± *se* | *s.* |
| 'Cacanska Bestrna' | T1 | **1020.62** | 35.29 | a |
| | T2 | **912.14** | 13.52 | a |
| | T3 | **1061.32** | 26.32 | a |
| | T4 | **1076.07** | 63.21 | a |
| | T5 | **1059.94** | 40.01 | a |
| | T6 | **1071.39** | 63.74 | a |
| | | | | |
| 'Loch Ness' | T1 | **1379.27** | 59.85 | a |
| | T2 | **1411.50** | 112.50 | a |
| | T3 | **1400.10** | 91.61 | a |
| | T4 | **1321.79** | 78.07 | a |
| | T5 | **1320.41** | 105.39 | a |
| | T6 | **1339.10** | 16.39 | a |
| | | | | |
| 'Navaho' | T1 | **1134.70** | 30.67 | a |
| | T2 | **1217.76** | 17.85 | a |
| | T3 | **1206.84** | 27.84 | a |
| | T4 | **1139.63** | 27.12 | a |
| | T5 | **1171.55** | 64.89 | a |
| | T6 | **1161.72** | 49.70 | a |
| | | | | |
| 'Smoothstem' | T1 | **1061.56** | 37.07 | c |
| | T2 | **1207.05** | 114.82 | bc |
| | T3 | **1609.71** | 117.33 | a |
| | T4 | **1222.53** | 90.76 | bc |
| | T5 | **1436.78** | 76.37 | ab |
| | T6 | **1330.09** | 113.90 | ab |
| | | | | |
| 'Thornfree' | T1 | **1224.18** | 37.88 | d |
| | T2 | **1410.87** | 52.94 | c |
| | T3 | **1580.36** | 36.05 | ab |
| | T4 | **1480.08** | 56.29 | bc |
| | T5 | **1652.95** | 28.15 | a |
| | T6 | **1567.09** | 78.94 | abc |
| | | | | |
| Cultivar | 'Cacanska Bestrna' | **1032.75** | 21.80 | E |
| | 'Loch Ness' | **1362.03** | 31.20 | B |
| | 'Navaho' | **1172.03** | 15.74 | D |
| | 'Smoothstem' | **1261.29** | 32.29 | C |
| | 'Thornfree' | **1485.92** | 34.55 | A |
| | *p* term | **0.0000*** ** | | |
| | *p* cultivar | **0.0000*** ** | | |
| | *p* INT | **0.0127*** | | |

Different small letters (a-d) in columns denote statistically significant differences between sampling dates for each blackberry cultivar by Duncan's multiple range test (p<0.05). Different capital letters (A-E) in columns denote statistically significant differences by Duncan's multiple range test (p < 0.05) among different cultivars. (Asterisk: *- statistically significant differences at *p*-Value <0.05; ** - statistically significant differences at *p*-Value <0.001; *** - statistically significant differences at *p*-Value <0.0001, no asterisk – statistically non-significant). Harvest date: T1- 28.07; T2-4.08; T3-10.08; T4-18.08.; T5-25.08; T6-1.9.2015.
